# Blood making: learning what to put into the dish

**DOI:** 10.12688/f1000research.21245.1

**Published:** 2020-01-23

**Authors:** Ana G Freire, Jason M Butler

**Affiliations:** 1 Center for Discovery and Innovation, Hackensack University Medical Center, Nutley, USA; 2 Molecular Oncology Program, Georgetown University, Washington D.C., USA

**Keywords:** hematopoietic stem cell, AGM, hemogenic endothelium, pluripotent stem cell

## Abstract

The generation of hematopoietic stem cells (HSCs) from pluripotent stem cell (PSC) sources is a long-standing goal that will require a comprehensive understanding of the molecular and cellular factors that determine HSC fate during embryogenesis. A precise interplay between niche components, such as the vascular, mesenchymal, primitive myeloid cells, and the nervous system provides the unique signaling milieu for the emergence of functional HSCs in the aorta-gonad-mesonephros (AGM) region. Over the last several years, the interrogation of these aspects in the embryo model and in the PSC differentiation system has provided valuable knowledge that will continue educating the design of more efficient protocols to enable the differentiation of PSCs into *bona fide*, functionally transplantable HSCs. Herein, we provide a synopsis of early hematopoietic development, with particular focus on the recent discoveries and remaining questions concerning AGM hematopoiesis. Moreover, we acknowledge the recent advances towards the generation of HSCs *in vitro* and discuss possible approaches to achieve this goal in light of the current knowledge.

## Introduction

Throughout the lifespan of an organism, the supply of blood cells is guaranteed by hematopoietic stem cells (HSCs) that replenish lineage-restricted progenitors while maintaining their stem cell pool. Because these cells are capable of sustaining the entirety of the blood hierarchy, they constitute a cellular source for transfusion, immunotherapies, and transplantation. HSC transplantation constitutes a crucial therapy for hematological disorders. However, the supply for patients is insufficient owing to shortage of histocompatible donors and limited cell number. Thus, the ability to generate HSCs *in vitro* will provide access to an unlimited number of cells that are capable of long-term reconstitution of all blood cell lineages. The derivation of embryonic stem cells (ESCs) and, subsequently, induced pluripotent stem cells (iPSCs) has provided the platforms to, in theory, derive HSCs given their intrinsic property to generate derivatives of the three germ layers. Yet, despite innumerous efforts, the generation of HSCs from non-genetically manipulated mouse or human pluripotent stem cells (hPSCs) has still not been successful. A full understanding of how HSC fate is specified during embryo development is crucial, as it will provide useful cues to educate the design of *in vitro* differentiation protocols. The inefficacy in doing so thus far is related to the inherent complexity of the hematopoietic developmental process and the incomplete identification of all the critical factors that make HSC specification possible in the embryo. Comprehension of the initiating events underlying the emergence of the distinct hematopoietic waves endowed with different lineage potential as well as of the microenvironmental cues that instruct and nurture HSC emergence, maturation, and expansion is fundamental. In the following sections, we will revisit the knowledge in early hematopoietic development, with particular focus on the events occurring in the aorta-gonad-mesonephros (AGM) region, the location where HSCs are first detected in the embryo, from the origin of the HSC precursor to the molecules and niche components that guide HSC fate. Lastly, we will review recent advances in the attempt to generate long-term HSCs in the dish and the important contributions these studies provided to get closer to the Holy Grail of the hematopoietic field.

## Early hematopoietic development at a glance

Blood formation is a conserved process among vertebrates that occurs in several waves, contributing different hematopoietic cell types in the yolk sac (extraembryonic) and within the embryo proper (intraembryonic). The first hematopoietic wave, called primitive hematopoiesis, emerges within the yolk sac blood islands around embryonic day (E) 7.0–7.5 in the mouse. Hematopoietic differentiation at this stage is limited to cells of the erythroid, macrophage, and megakaryocytic lineages and serves the needs of a rapidly growing embryo ^1^. Following the formation of primitive cells, a second wave contributes with erythro-myeloid progenitors (EMPs) as well as lymphomyeloid, B-1, and T cell-restricted progenitors before the emergence of HSCs ^2– 6^. Because the first and second waves arise before the first adult-type HSCs are detected in the embryo, they are considered to be HSC-independent hematopoiesis ^1, 7^. Interestingly, derivatives of the HSC-independent hematopoiesis persist to adult life, such as the microglia in the brain and the tissue-resident macrophages found in several organs ^8, 9^. The existence of long-lived functional blood cells that did not derive from an HSC challenges the classical hierarchical model of adult hematopoiesis. The hallmark of an adult HSC is its capacity of long-term, multilineage reconstitution in adult recipients in a setting of serial transplantation ^10^. This definition set the foundation that HSC emergence begins around E10.5 in the dorsal aorta (DA) and vitelline/umbilical arteries of the mouse embryo ^11– 14^. Nascent HSCs are present within intra-aortic clusters in close contact with endothelial cells (ECs) lining the floor of the DA ^14^. In the human, intra-aortic clusters are detected around 27 days of gestation ^15^, with HSC long-term repopulating activity being demonstrated by transplantation assay into immunodeficient mice ^16^.

**Figure 1.**
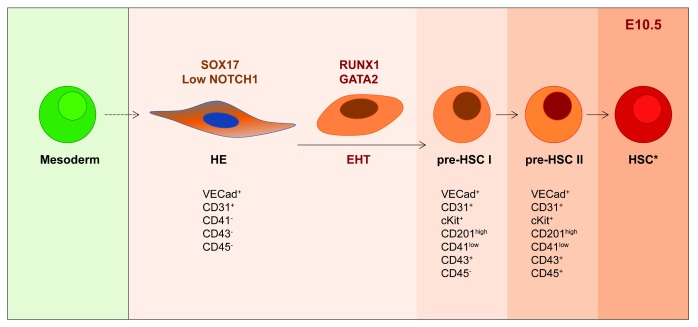
Representation of the consensually accepted sequential steps of differentiation to adult-type HSCs in the AGM. HSCs derive from a precursor that descends from mesoderm and expresses endothelial genes, called hemogenic endothelium (HE). HE undergoes endothelial hematopoietic transition (EHT) and maturation to functional HSCs through intermediate HSC precursors (type I and II pre-HSCs). During this process, a precise regulation of the arterial-associated genes *Notch1* and *Sox17* and of the critical players in EHT, *Runx1* and *Gata2*, takes place. Moreover, hematopoietic markers (i.e. ** c-Kit, CD41, CD43, and CD45) are upregulated while genes of endothelial affiliation (i.e. VE-Cadherin and CD31) are still expressed ^24– 28^. *HSCs are believed to display a similar phenotypic signature to type II pre-HSCs, but their function is demonstrated by transplantation into adult irradiated recipients. AGM, aorta-gonad-mesonephros; E, embryonic day; HSC, hematopoietic stem cell.

Observations made 100 years ago led to the hypothesis that blood cells emerging in the DA possess an endothelial origin ^17, 18^. More recent studies have provided compelling evidence, through live imaging and lineage tracing, for the existence of a specialized vascular endothelium, the denominated hemogenic endothelium (HE), that undergoes endothelial hematopoietic transition (EHT) ^19– 23^. Maturation towards HSC fate occurs through a multi-step differentiation process from hematopoietic precursors (type I and II pre-HSCs) that express genes associated with the endothelial lineage, such as *Cdh5* (or *VEcad*) and *Pecam1* (or *Cd31*) ( Figure 1) ^24– 28^. Later, it was discovered that HE is not restricted to the developing embryonic vasculature where HSCs emerge, existing also in the yolk sac where it serves as a precursor of primitive erythrocytes ^29^ and EMPs ^30^ as well as B ^6^ and T cells ^5^ prior to HSC emergence.

The emergence of HSCs in the DA is a highly dynamic and transient phenomenon that declines around E12.5 in the mouse, coinciding with the entrance of HSCs in the bloodstream and seeding of the fetal liver, which will support HSC expansion prior to migration to the fetal spleen and bone marrow ^28, 31^. Additional sources where the presence of HSCs has been reported (e.g. the placenta, head, and yolk sac) were suggested to also contribute to the HSC pool in the fetal liver ^32– 36^. However, the capacity of these sources to originate HSCs *de novo*, rather than promoting their expansion, remains a controversial topic which is greatly hampered by the onset of circulation around E8.25 ^35, 37– 39^. Recently, the *de novo* generation of hematopoietic stem/progenitor cells (HSPCs) from HE existing in the bone marrow of late fetus/young adult chickens and mice has also been suggested ^40^.

Studies using limiting-dilution transplantation assays have estimated that the AGM contains very few HSCs (one to three) and proposed that the rapid increase in HSC number in the fetal liver could be the result of maturation of a large pool of pre-expanded pre-HSCs generated in the AGM region ^28, 32^. Recent reports utilizing multicolor fate mapping in zebrafish and mouse models have suggested that the number of hematopoietic precursors emerging at the AGM, and contributing to adult hematopoiesis, is significantly higher than initially predicted ^41, 42^. The functional readout of HSCs autonomously generated from the AGM has been facilitated by the utilization of platforms that allow *ex vivo* maturation and/or expansion, such as organ explant culture ^12, 32, 43^, co-aggregation culture with OP9 stromal cells ^24, 44^, or co-culture with Akt-expressing AGM-derived ECs ^45, 46^. It is not until E11.5 that functional AGM-derived HSCs can be robustly detected by direct transplantation into adult recipients, likely because of the achievement of a critical number of HSCs ^24^. The fact that hematopoietic precursors are able to acquire functional properties after a period of *ex vivo* co-culture with niche cells suggests that these *in vitro* platforms are able to provide the cues encountered within the embryonic environment. This anticipates that it might be possible to recapitulate *in vitro* the process of HSC generation if we recreate the complex interactions within the stem cells’ niche.

## AGM: the cradle of HSCs

The observation that HE located in the DA and vitelline/umbilical arteries originates HSCs highlights the importance of comprehending the combination of spatiotemporal signals and niche cells that compose the AGM region. Curiously, HE giving rise to functional HSCs is detected only within the large arteries of the embryo, whereas HE in the yolk sac is associated with both arterial and venous endothelium ^13, 30, 47^. This evidence, combined with the observation of direct hematopoietic cell emergence from aortic endothelium, led to the hypothesis that acquisition of arterial identity precedes HSC emergence. The origin of the aortic HE and whether it shares a common lineage with arterial endothelium is still a topic of debate. The loss of critical regulators of arterial identity, such as SOX17 and NOTCH1 ^48– 50^, is detrimental for HSC generation or maintenance in the mouse embryo ^51, 52^. However, a tight control of the activity of these pathways is required to ensure proper HSC specification, with downregulation being necessary during formation of the hematopoietic clusters ^53, 54^. RUNX1 and GATA2 are also indispensable players during EHT ( Figure 1) in the zebrafish, chick, and mouse AGM and during ESC differentiation ^23, 55– 60^, whose timing and duration of intervention are tightly controlled ^61, 62^. Nevertheless, studies that have reported the presence of hematopoietic precursors in the subendothelial aortic layers in the mouse AGM region added complexity to our understanding of the origin of the HE ^24, 63^. In addition, it has been recently shown in the chicken that the coelomic epithelium-derived mesenchyme invades through the ventral wall of the DA prior to formation of the intra-aortic clusters ^64^. Studies in hPSCs have identified HE and non-HE lineages during *in vitro* differentiation ^65^ and have suggested that the HE and the precursors of the arterial vascular endothelium represent distinct lineages deriving from hPSC-derived mesodermal progenitors ^66^. These observations during hPSC *in vitro* differentiation led to a proposed model of human HE development, in which HE in the subaortic mesenchyme migrates to the AGM region to integrate with the arterial ECs within the ventral wall of the DA ^67^. Later studies in hPSCs suggested that, after specifying from mesodermal progenitors, HE can be specified into an arterial-type HE with lymphoid and myeloid potential *in vitro* through overexpression of *Ets1* or modulation of MAPK/ERK signaling in a process dependent on Notch signaling ^68, 69^. Recently, RNA-sequencing studies in the mouse embryo revealed that the aortic HE transcriptome is very similar to that of arterial ECs of the DA ^26, 70^ and distinct from the yolk sac HE ^70^. Additionally, a recent single-cell RNA-sequencing study in human AGM reported that the HE displays an arterial gene signature ^71^. Moreover, a study in zebrafish using a BAC *runx1* transgenic reporter showed that *runx1* expression in HE mediates silencing of arterial endothelial genes, including *dll4*, while *runx1 ^–/–^* mutants maintain arterial identity ^72^. This finding reconciles the previous demonstration that cells in the mouse DA that experience high levels of NOTCH1 activity through DLL4 are committed to arterial EC fate, whereas specification of HSCs depends on low levels of NOTCH1 enabled by JAGGED1 ^73^. Furthermore, it suggests the aortic endothelium is the precursor of HE ^72^, in line with earlier evidence provided by live imaging and lineage tracing studies in the chick, zebrafish, and mouse embryo ^19, 21– 23, 74^.

An important observation is that the emergence of intra-aortic clusters is located at the ventral wall of the DA in the chick, zebrafish, and human AGM ^15, 19, 21, 23^. In the mouse, although mostly ventralized, some clusters are also found along the dorsal wall ^22, 75, 76^. Yet repopulating activity is predominantly restricted to intra-aortic clusters localized ventrally in the DA ^75^. A plausible explanation might be that ventral aortic ECs, conversely to dorsal ECs, derive from splanchnopleural mesoderm, which is endowed with hematopoietic potential ^77^. The dorsoventral polarity of HSC function correlates with asymmetric signaling in the embryonic DA, with polarized Sonic Hedgehog (SHH) and bone morphogenetic protein 4 (BMP4) signaling and Kit ligand (KITL) expression being reported ^78– 80^. Reinforcing the transient nature of AGM hematopoiesis, a precise control of BMP activity has been shown in mouse and zebrafish in order to restrict HSC formation and allow maturation ^80– 82^. In the zebrafish, fibroblast growth factor (FGF) signaling was found to play a role in modulating the BMP pathway ^82^. Moreover, Tenascin C, an extracellular matrix glycoprotein that facilitates cell-to-cell interactions and cell migration, is also abundant in the mesenchymal tissue underneath the ventral wall of the human DA ^83^. A posterior study in hPSCs correlated the high production of Tenascin C in overconfluent OP9 stromal lines with the ability to induce higher hematopoietic potential ^84^.

**Figure 2.**
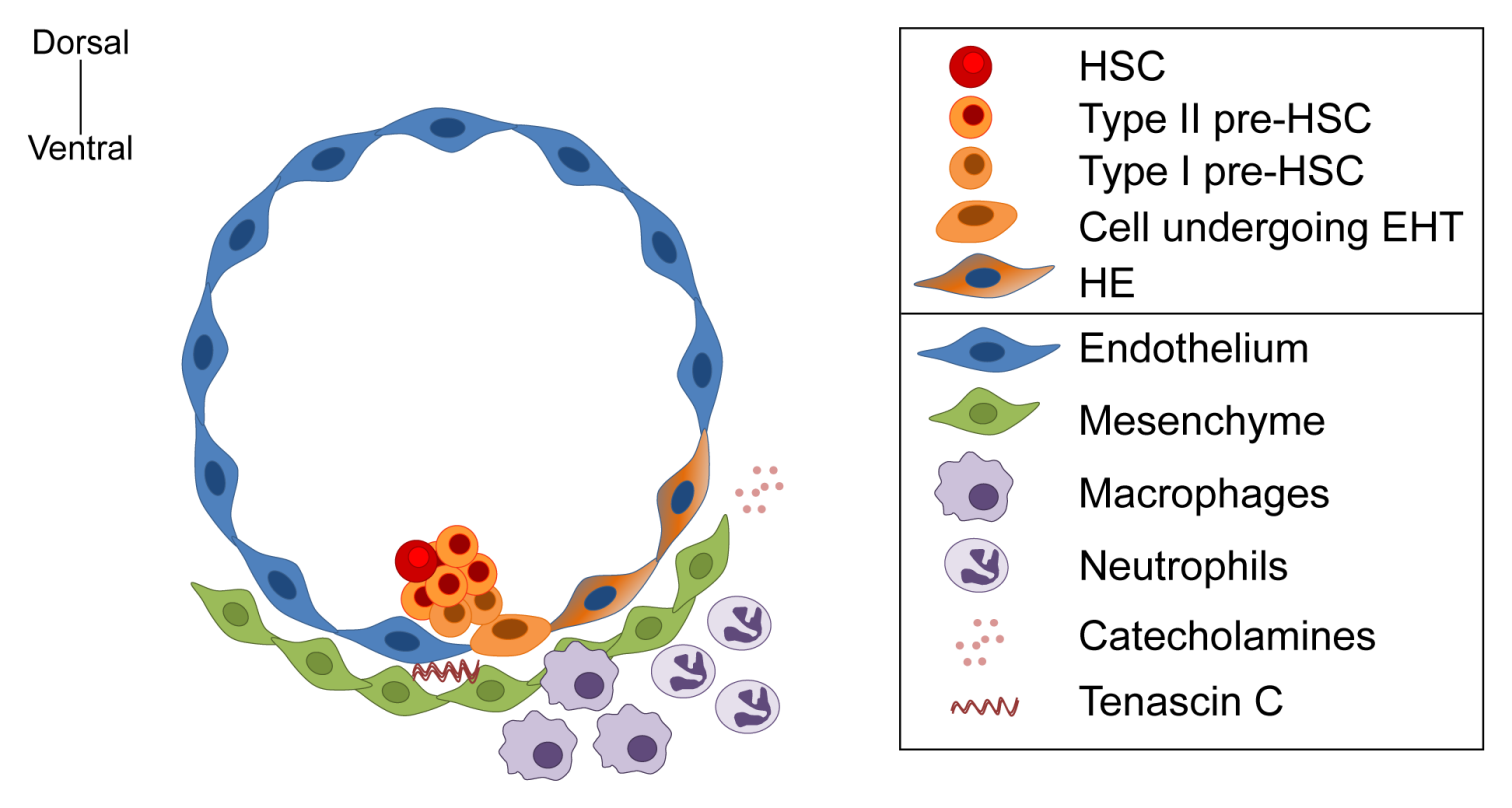
Representation of niche components that participate during HSC emergence in the AGM. A precise interplay between niche components, including ECs, mesenchymal cells, primitive myeloid cells, and components of the nervous system, provides the unique signaling milieu for the emergence of HSCs in the AGM region. AGM, aorta-gonad-mesonephros; EC, endothelial cell; EHT, endothelial hematopoietic transition; HSC, hematopoietic stem cell.

Contributing to the highly dynamic nature of the AGM, a complex crosstalk between different niche components, including the recruitment of different cellular and molecular factors to the vicinity of the DA, takes place during HSC emergence ( Figure 2). The subaortic mesenchyme contributes to HSC formation in the chicken embryo by inducing RUNX1 expression in the ventral aortic endothelium ^58^. Moreover, studies suggested a link between the developing nervous system and enhancement of HSC emergence, as shown by the inductive effect of catecholamines in the mouse ^85^ and neuronal serotonin in the zebrafish ^86^. Interestingly, from the yolk sac-derived wave, macrophages ^87, 88^ and neutrophils ^89^ contribute with local sterile inflammatory signaling that promotes HSC emergence. Evidence in the zebrafish further demonstrated that tumor necrosis factor alpha (TNFα) signaling produced by neutrophils triggers JAGGED1-mediated Notch signaling in the aortic endothelial layer, thereby promoting HSC specification ^89^. Furthermore, macrophages establish a close interaction with the emerging intra-aortic hematopoietic cells in zebrafish, human ^90^, and mouse AGM ^88^ and remodel the extracellular matrix, thus permitting HSC mobilization to the caudal hematopoietic tissue (analogous to the mammalian fetal liver in the zebrafish) ^90^. Importantly, HSC repopulating activity was impaired when AGM explants were depleted of macrophages, thus indicating macrophages are crucial for the generation and/or maturation of functional HSCs in the mouse AGM ^88^. In addition, quail/chick orthotopic transplantations have shown that somite-derived ECs contribute distinct waves, first to build the roof of the DA and later to replace the hemogenic floor ceasing hematopoietic generation in the ventral aortic wall ^91^. In the zebrafish, somite-derived ECs integrate the DA while contributing to HSC formation by eliciting inductive signals ^92^. Though phenotypic HSC precursors are found in both subluminal and luminal layers, HSCs are localized exclusively within the endothelial aortic layer ^24, 44^, strongly suggesting that the final step of maturation into functional HSCs needs instructive cues from the non-hemogenic vascular niche. Accordingly, hematopoietic precursors are able to expand and mature into functional HSCs after *in vitro* co-culture with Akt-expressing AGM-derived EC lines ^45^. This could be in part explained by the secretion of KITL by aortic ECs ^80, 93^, a key regulator driving pre-HSC/HSC survival and maturation in the AGM region ^25, 80, 93^. Moreover, *Tie2‐Cre::* *Kitl* ^*Δ/Δ*^ mice, where *Kitl* is specifically deleted in the endothelium, showed decreased numbers of phenotypic pre‐HSC II/HSCs in the AGM at E11.5, further resulting in fewer reconstituted mice upon transplantation ^93^. The instructive role of the vascular niche in adult HSC function in the bone marrow, including the supply of KITL, has been extensively demonstrated ^94^. The full understanding of these complex interactions and the identification of all the moving parts that take place in AGM hematopoiesis will help us design protocols that aim to derive HSCs from PSCs.

## The quest for generating HSCs in the dish

Historically, hematopoietic development *in vitro* recapitulates the patterns observed in the embryo, following a step-wise differentiation toward induction and patterning of the mesoderm and commitment to the distinct hematopoietic waves ^67^. Although differentiation of PSCs to multiple blood cell types is feasible, the generation of *bona fide* blood stem cells is still a challenge. Strategies utilizing genetic manipulation of mouse ESC-derived hematopoietic progenitors, such as transduction with *Hoxb4,* reported long-term engraftment in irradiated adult hosts though underperformance in terms of lymphoid contribution ^95^. More recently, a study in non-genetically manipulated mouse ESCs has provided exciting evidence that it might be possible to obtain cells with engrafting ability within a transient time window during differentiation. However, engraftment shown in immunodeficient mice declined over time, and demonstration of repopulation in secondary recipients was not provided ^96^. Despite the innumerous efforts to generate HSCs from human PSCs, studies that have performed transplantation into immunodeficient mice have reported low engraftment efficiency and/or lacked proof of robust lymphoid contribution ^97– 100^. Noteworthy, a study in hPSCs utilizing a differentiation protocol that enhances the expression of *HOXA* genes, shown to be downregulated in PSC-derived hematopoietic progenitors when compared to fetal liver HSPCs and cord blood CD34 ^+^ cells ^101, 102^, was able to recapitulate an AGM-like vessel network. Yet hematopoietic cells derived using this protocol were not capable of long-term engrafting ability after transplantation ^102^.

Nonetheless, studies using PSCs have contributed valuable information on early cell fate determination, such as the identification of signals that repress specification into primitive hematopoiesis (i.e. inhibition of ACTIVIN-NODAL signaling and activation of WNT during early stages of differentiation) ^102– 104^. Moreover, observations in this *in vitro* system suggest that an early mesoderm-derived HE specifies into distinct subsets that differ in the hematopoietic lineage potential ^68, 69, 105, 106^. In the attempt to foster hematopoietic instruction by providing niche signals, some studies have established co-cultures with human umbilical vein endothelial cells ^97, 106^ or AGM-derived stromal cell lines ^98, 107^. The growing knowledge on the molecular and cellular factors that instruct HSC generation and maturation will be crucial for the implementation of efficient protocols to derive HSC fate *in vitro*.

## Conclusions

The ability to generate *bona fide* blood stem cells from PSCs has been intensively investigated given the impact it would have in clinical applications. The challenges are related not only to the incomplete knowledge of all the critical factors important for HSC emergence but also to the inherent complexity of the hematopoietic developmental process. In light of the understanding that erythroid-myeloid and B and T lymphoid potential is detected prior to repopulating capacity, it becomes unclear whether hematopoietic cells obtained during PSC differentiation, including T cells, represent the *in vitro* counterpart of the HSC-independent hematopoiesis occurring in the embryo. Because of the obstacles posed by direct differentiation *in vitro*, several laboratories have made efforts to achieve this goal by using the reprogramming strategy. Recent studies have reported the generation of hematopoietic cells with long-term repopulating ability and immune competence ^108, 109^. Towards a safer approach, the successful generation of HSCs from reprogramming methods that do not require transgene integration will need to be tested. The generation of HSCs in the dish, by exposing PSCs to signaling cues that mimic the embryo environment, would be the safest approach. The challenge here is to identify the combination of soluble factors and inhibitors and the niche components required to drive specification of HSC fate. The recent discoveries documenting new cell types and signaling molecules that participate during AGM hematopoiesis added valuable information to the field and will help to establish *in vitro* differentiation protocols. The current knowledge supports the idea that the aortic endothelium is pivotal for HSC emergence. A specialized hemogenic endothelium, in a complex crosstalk with other cell types, undergoes EHT mediated by the interplay of several transcriptional regulators that act transiently to specify hematopoietic fate. Simultaneously, non-hemogenic ECs provide instructive cues that enable the generation of functional HSCs. Thus, to mimic the transient nature of AGM hematopoiesis, a fine tuning of the signaling milieu will have to take place to ensure progression of EHT and generation of functional HSCs. Importantly, given the evidence that several niche components are essential for HSC emergence and maturation, the niche cells need to be included in the equation. This could be attempted either by direct co-culture or by supplementing the culture with key factors provided by the niche cells. Moreover, provided that HSC emergence is achieved, culture conditions will need to support maturation and expansion to numbers that can robustly engraft adult recipients in a transplantation setting, the only reliable readout assay to confirm the presence of functional long-term HSCs. The ability to expand long-term reconstituting HSCs from adult bone marrow or umbilical cord blood (UCB) sources *ex vivo* has also demonstrated challenges. Earlier attempts have established *in vitro* culture conditions that did not support the expansion to relevant cell numbers and/or caused adverse effects on HSPCs that resulted in improper differentiation ^110^. More recently, the incorporation of the small molecules SR-1 ^111^ and UM171 ^112^ or valproic acid ^113^ or direct co-culture with ECs ^114^ to serum-free media supplemented with cytokines has shown robust levels of CD34 ^+^-UCB expansion with long-term repopulating ability. The *ex vivo* vascular niche platform has also been shown to support the expansion of engraftable adult bone marrow-derived CD34 ^+^ cells ^115^. Interestingly, a protocol that enables long-term *ex vivo* culture of mouse adult bone marrow HSCs that expand robustly and maintain their functionality has been reported recently ^116^. Proven the robust generation and expansion of *bona fide* blood stem cells by direct differentiation from PSCs *in vitro*, the Holy Grail in the field will be ultimately achieved, providing not only an *in vitro* platform to model development and hematological diseases but also an unlimited source of cells for clinical applications.

## Abbreviations

AGM, aorta-gonad-mesonephros; BMP4, bone morphogenetic protein 4; DA, dorsal aorta; EC, endothelial cell; EHT, endothelial hematopoietic transition; EMP, erythro-myeloid progenitor; ESC, embryonic stem cell; FGF, fibroblast growth factor; HE, hemogenic endothelium; hPSC, human pluripotent stem cell; HSC, hematopoietic stem cell; HSPC, hematopoietic stem/progenitor cell; iPSC, induced pluripotent stem cell; Kitl, Kit ligand; PSC, pluripotent stem cell; SHH, Sonic Hedgehog; TNFα, tumor necrosis factor alpha; UCB, umbilical cord blood.
